# Repeat and Redo Microdissection Testicular Sperm Extraction (Micro-TESE) in Non-Obstructive Azoospermia: A Narrative Review

**DOI:** 10.3390/jcm15208088

**Published:** 2026-10-18

**Authors:** Erriketi Douvli, Athanasios Zachariou, Ioannis Giannakis, Sotirios Koukos, Aris Kaltsas, Harikleia Maria Sofikiti, Michail Baltogiannis, Fotios Dimitriadis, Suichi Morizane, Nikolaos Sofikitis

**Affiliations:** 1 Urology Department, Ioannina University, 45500 Ioannina, Greece; edouvli94@gmail.com (E.D.);; 2 3rd Urology Department, Athens University, 12462 Athens, Greece; 3 1st Urology Department, Aristotle Thessaloniki University, 54635 Thessaloniki, Greece; 4 Division of Urology, Department of Surgery, Faculty of Medicine, Tottori University, Yonago 683-8504, Tottori, Japan

**Keywords:** non-obstructive azoospermia, microdissection testicular sperm extraction, redo micro-TESE, repeat micro-TESE, sperm retrieval rate, hormonal therapy, antioxidants

## Abstract

Microdissection testicular sperm extraction (micro-TESE) is the standard surgical approach for sperm retrieval in men with non-obstructive azoospermia (NOA). However, further procedures may be required either after successful retrieval to support additional intracytoplasmic sperm injection cycles or as salvage treatment following initial failure. This narrative review evaluates outcomes of repeat micro-TESE after previous success and redo micro-TESE after failure, focusing on sperm retrieval rates (SRRs), predictors of success, and strategies proposed to optimize outcomes before reoperation. Repeat procedures following successful retrieval generally show high SRRs, with one large series reporting rates of 73.9–92.5% at subsequent attempts; however, these estimates reflect progressively selected populations and should not be interpreted as independent per-attempt probabilities. In contrast, redo micro-TESE after initial failure achieves sperm retrieval in approximately 10–21% of cases in most series, although higher rates have occasionally been reported. Previous retrieval outcome and testicular histopathology appear most informative for counselling, whereas serum FSH, testicular volume, genetics, and the interval between procedures have inconsistent predictive value. Hormonal pretreatment may modify the intratesticular environment, but evidence for improved redo retrieval remains limited and heterogeneous. Antioxidant therapy is biologically plausible but has not been adequately evaluated in this setting. Technical factors, including the adequacy of the initial procedure, surgical expertise, and laboratory processing, may also influence outcomes. Prospective controlled studies with standardized reporting are needed to define optimal patient selection and management before repeat or redo micro-TESE.

## 1. Introduction

Non-obstructive azoospermia (NOA) represents the most severe form of male infertility and is defined by the complete absence of spermatozoa in the ejaculate on two separate semen analyses [1,2]. NOA accounts for approximately 60–70% of azoospermia cases, affects an estimated 10–15% of infertile men, and occurs in approximately 1% of men in the general population [3,4]. Its aetiology is heterogeneous and includes genetic abnormalities, such as Klinefelter syndrome (KS) and Y-chromosome microdeletions [5], cryptorchidism, testicular torsion or trauma, infectious orchitis, gonadotoxic exposure from chemotherapy or radiotherapy, and varicoceles [6]. Nevertheless, the underlying cause remains idiopathic in nearly half of affected men [3].

Since its introduction by Schlegel [7], micro-TESE has become the gold-standard surgical technique for sperm retrieval in men with NOA. Compared with conventional testicular sperm extraction (c-TESE) and testicular sperm aspiration (TESA), micro-TESE achieves higher sperm retrieval rates (SRRs) while minimizing tissue excision and reducing the risks of vascular injury and postoperative testicular dysfunction [1,8]. Reported SRRs at first-attempt micro-TESE generally range from approximately 40% to 63% [2,8,9]. In a systematic review and meta-analysis of 15 studies involving 1890 men with NOA, sperm retrieval was 1.5 times more likely with micro-TESE than with c-TESE (95% CI: 1.4–1.6) and 2.0 times more likely with c-TESE than with TESA (95% CI: 1.8–2.2) [10].

Despite these advantages, a substantial proportion of men with NOA have unsuccessful sperm retrieval at their first micro-TESE. Conversely, even after successful retrieval, couples may require additional intracytoplasmic sperm injection (ICSI) cycles and therefore further sperm retrieval procedures when an adequate supply of cryopreserved sperm is unavailable. Two clinically distinct scenarios consequently arise: (i) repeat micro-TESE, performed after a previously successful sperm retrieval, usually to obtain sperm for subsequent ICSI cycles, and (ii) redo (salvage) micro-TESE, performed after an initially unsuccessful retrieval [3]. The distinction is clinically important because these scenarios differ substantially in prognosis and in the considerations that inform patient counselling and selection for further surgery. Moreover, each additional testicular procedure carries potential structural and endocrine consequences, including cumulative tissue loss and a possible further decline in testosterone production, as well as psychological and financial burdens for patients and couples [11]. Sexual function is a further consideration, since repeated testicular surgery and any accompanying decline in androgen production may adversely affect erectile function, libido, and sexual satisfaction [12].

These concerns have generated considerable interest in strategies that might optimize the likelihood of sperm retrieval before another surgical attempt. Among the most widely discussed are hormonal optimization, aimed at improving the intratesticular hormonal environment and supporting residual spermatogenesis, and antioxidant supplementation, intended to mitigate oxidative stress potentially contributing to testicular dysfunction in NOA.

The aim of this narrative review is to critically evaluate the available evidence on repeat micro-TESE after a previous successful sperm retrieval and on redo micro-TESE after an initially failed attempt in men with NOA. Particular emphasis is placed on sperm retrieval outcomes, factors associated with successful reoperation, and strategies proposed to optimize the likelihood of sperm retrieval before a subsequent procedure, with a specific focus on hormonal and antioxidant therapy. The review also examines the limitations of the current evidence and their implications for patient selection, counselling, and clinical decision-making regarding repeat or redo micro-TESE.

## 2. Methods: Literature Search Strategy

The present work is a narrative synthesis underpinned by a deliberately structured search of the literature. It was never intended to function as a systematic review or meta-analysis, and no attempt has therefore been made to pool effect estimates, to construct a PRISMA flow diagram, or to score the included studies against a formal risk-of-bias instrument.

MEDLINE (through PubMed), Web of Science and Scopus were interrogated for material appearing between January 2000 and May 2026. Those databases were supplemented in two ways: by directed searching in Google Scholar and by reading through the bibliographies of the clinical studies, reviews and guideline documents that met our criteria, so that older or less well indexed reports were not lost. Older publications were identified through backward citation searching and retained to provide foundational surgical and histopathological background. One axis of the search described the population and the operation—non-obstructive azoospermia alongside the redo, repeat and second-look variants of micro-TESE. A second axis described the interventions and outcomes of interest and covered sperm retrieval rate, testicular histopathology, testicular volume, hormonal stimulation, human chorionic gonadotropin, follicle-stimulating hormone, aromatase inhibitors, selective oestrogen receptor modulators, testosterone, hypogonadism, Leydig cells, antioxidants, oxidative stress and varicocelectomy.

A report qualified as direct clinical evidence if its participants were adult men with NOA proceeding to a second or later surgical retrieval, and if it reported retrieval, reproductive or postoperative outcomes. Randomized trials sat alongside prospective and retrospective cohorts; publication in English was a practical requirement. Where a study described its treatment protocol and intervention sequence in enough detail to be compared with others, that report carried greater weight in the synthesis. Several categories fell outside this definition and were not counted as direct evidence: series restricted to obstructive azoospermia, paediatric cohorts, animal and bench work, non-surgical interventions reported without any micro-TESE endpoint, and technical descriptions that stopped short of postoperative outcomes. Where two or more publications appeared to describe overlapping patients, only one was retained—whichever was the more recent or reported the fuller dataset.

The database searches identified 362 records. Removal of 251 duplicates left 111 records for title and abstract screening, during which 81 were excluded for failing to meet the eligibility criteria. Any disagreements regarding article eligibility were resolved through discussion and consensus between the two primary investigators. The remaining 30 reports were assessed in full text; four lacked suitable patient data, leaving 26 studies for inclusion. A provisional evaluation of the revised manuscript using the six-item SANRA instrument is presented in Supplementary Table S1. This assessment addresses the quality of the narrative review and does not substitute for an appraisal of bias in the individual studies.

The decision to use a narrative synthesis reflects the state of the literature rather than methodological preference. Across redo micro-TESE series, substantial variation exists in study design, patient selection, intervention, and outcome definitions. Pooling these heterogeneous data would therefore risk implying a level of precision that the underlying evidence cannot support. A narrative approach also allows clinical outcomes to be interpreted alongside mechanistic evidence, guideline recommendations, and expert opinion, which is appropriate for a question shaped by several interacting factors. Its limitation is that the resulting synthesis is interpretative rather than quantitative and should therefore be weighed accordingly. For transparency, several cited publications were authored or co-authored by members of the present author group. These sources are discussed alongside the wider literature, with their study designs, limitations, and applicability to repeat or redo micro-TESE considered explicitly.

## 3. Outcomes of Repeat Micro-TESE After a Successful First Attempt

One of the largest and most detailed datasets on repeat micro-TESE comes from Ghalayini et al. [13], a retrospective series of 134 men with NOA who underwent 323 micro-TESE procedures between January 2013 and February 2020; consistent with the authors’ policy, most repeat procedures followed a previously successful sperm retrieval. Sperm was retrieved in 236 out of 323 procedures (73.1%). Observed SRRs in the progressively selected groups were 60.4% at the first procedure (81/134), 73.9% at the second (65/88), 86.9% at the third (53/61), and 92.5% at the fourth (37/40). The progressive increase reflects progressively selected populations enriched for previous retrieval success and must not be quoted as the per-attempt prognosis for an unselected cohort. These conditional rates do not demonstrate a benefit from increasing the number of operations. Success at each attempt was strongly associated with the outcome of the preceding procedure: a previously successful retrieval was associated with 10.1-fold higher odds of success at the second attempt, 5.6-fold at the third (relative to the outcome of the second procedure), and 16.5-fold at the fourth (relative to the outcome of the third). The interval between the first and second procedures also influenced outcome, each additional month being associated with a 1.3-fold increase in the odds of successful retrieval at the second attempt; the interval was no longer a significant predictor at subsequent attempts.

This pattern is supported by several earlier, smaller series. Talas et al. [14] followed 68 men with NOA and reported an SRR of 64% (44/68) at the initial procedure. Twenty-four men proceeded to a second micro-TESE—19 after a positive and five after a negative first attempt—and spermatozoa were recovered in 19 out of these 24 (16/19 of the previously positive and 3/5 of the previously negative men). Sperm was also retrieved at all four third attempts and at the single fourth attempt, giving an overall success rate of 24 of 29 repeat procedures (82%). Friedler et al. [15] similarly reported that repeated TESE followed by ICSI could achieve successful outcomes in men with histologically defined NOA. Kamal et al. [16] reported successful repeat testicular sperm extraction in 91.5% of men whose initial procedure had been positive. Westlander et al. [17] demonstrated the feasibility of repeating testicular sperm aspiration in 34 men, up to a sixth attempt in one patient, although the small cohort and the absence of confirmed histopathology limit direct comparison with micro-TESE-specific studies. In a larger historical cohort of 628 men undergoing up to six TESE attempts, Vernaeve et al. [18] likewise concluded that repeat TESE is associated with a high cumulative sperm recovery rate. These observations are consistent with the earlier findings of Tournaye et al. [19] regarding predictors of successful testicular sperm recovery.

Among the factors evaluated by Ghalayini et al. [13], testicular histopathology was the strongest predictor of successful sperm retrieval. Men with hypospermatogenesis had substantially higher SRRs than those with maturation arrest or Sertoli cell-only syndrome (SCOS): 94.8%, 40.9%, and 31.5%, respectively, at the first procedure, and 97.3%, 50.0%, and 59.5% at the second (both *p* < 0.001). By the third and fourth procedures, however, these differences were no longer statistically significant, SRRs having become uniformly high across histological groups. Larger testicular volume was independently associated with successful sperm retrieval at the first and second procedures, while lower FSH was associated with success only at the first. Neither factor remained significant at the third or fourth attempt. The authors suggested that this was due to selection bias, as men undergoing further procedures were more likely to have already shown features associated with successful retrieval.

**Figure 1:**
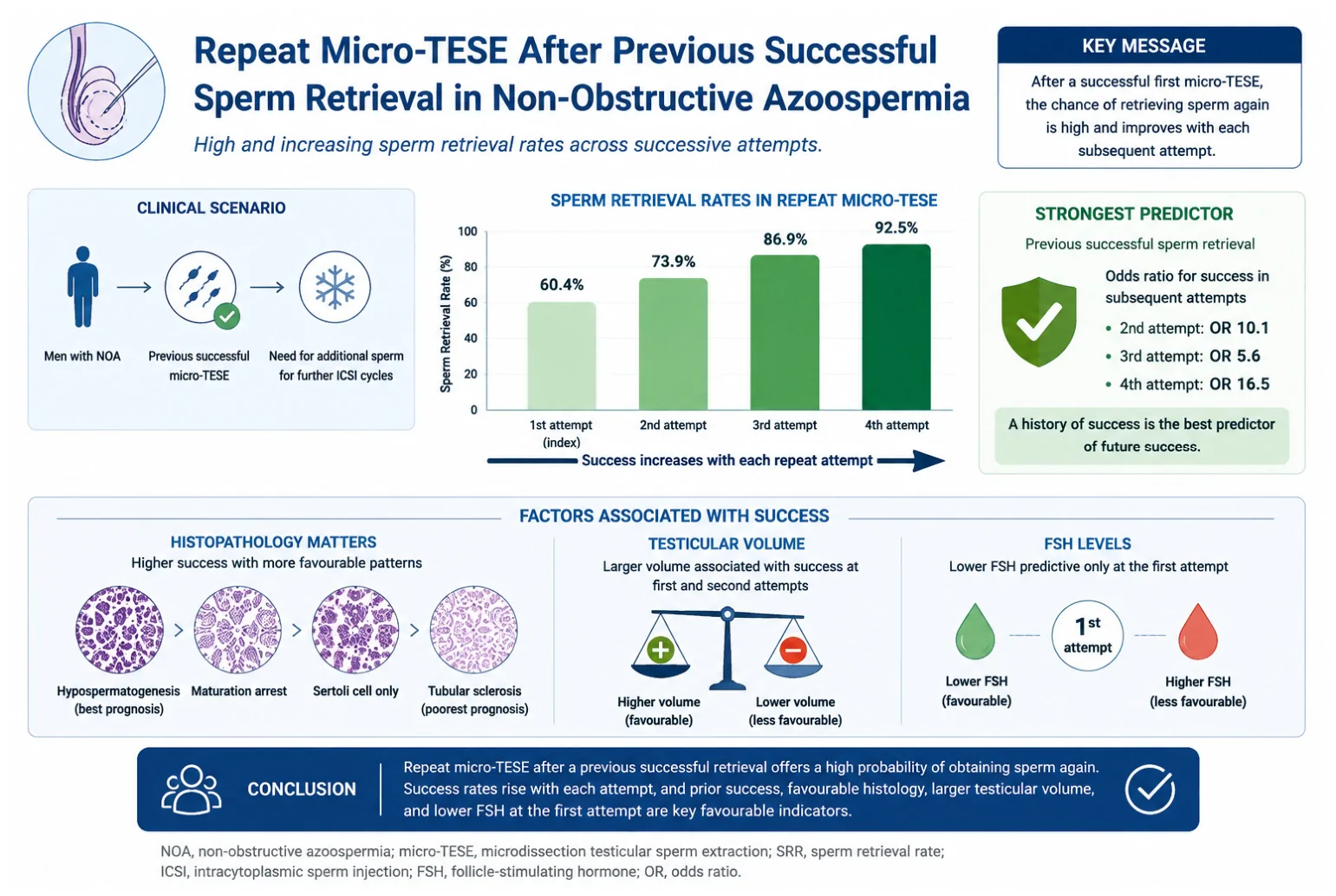
Observed sperm retrieval across successive micro-TESE attempts in the series of Ghalayini et al. [13]. Bars show retrieval in 81/134 (60.4%), 65/88 (73.9%), 53/61 (86.9%) and 37/40 (92.5%) procedures at the first through fourth attempts, respectively. Later groups were increasingly selected for previous successful retrieval. These conditional rates must not be quoted as per-attempt prognoses for an unselected cohort and do not demonstrate that additional operations improve retrieval probability. SRR, sperm retrieval rate.

The prognostic relevance of endocrine and histological characteristics is supported by other series. Al-Zubi et al. [20], evaluating preoperative hormonal parameters, testicular volume, and general health characteristics, found higher free testosterone levels and fewer previous micro-TESE attempts to be independent predictors of successful sperm retrieval. Kavoussi et al. [21] found that men with hypospermatogenesis had higher SRRs, pregnancy and live-birth rates, and were more likely to have enough sperm retrieved for multiple ICSI cycles than men with maturation arrest or SCOS. Overall, previous retrieval success, testicular histopathology, hormone levels, and the number of previous procedures should be considered when counselling men about repeat micro-TESE (Figure 1).

## 4. Outcomes of Redo Micro-TESE After an Initial Failure

The prognosis is considerably less favourable when the first micro-TESE has failed outright. In the narrative review by Elbardisi et al. [3], which synthesized nine original studies on redo micro-TESE following a negative first procedure (four retrospective and five prospective; interval between attempts: 6–24 months), most series reported successful sperm retrieval at the second attempt in only 10–21% of patients, although Yücel et al. [22] reported a higher rate of 42.8%. Reproductive outcomes were reported in only three out of the nine studies. Live births were also rarely reported: one each in the Haliloglu [23] and Shiraishi [24] cohorts, and four among six pregnancies in the FNA mapping study by Jarvis et al. [25]. This highlights the limited evidence on reproductive success even when sperm retrieval is successful.

A subsequent systematic review and meta-analysis by Zhang et al. [26] of redo micro-TESE after a failed initial c-TESE reached broadly consistent conclusions, describing modest but clinically meaningful pooled retrieval rates and identifying favourable histology, lower FSH, and a shorter interval since the index procedure as the most consistently reported predictors of redo success. In a multicentre cross-sectional study, Boeri et al. [27] likewise examined redo micro-TESE after failed classic TESE and emphasized that the surgical experience of the team performing the redo procedure may itself influence the outcome. Both analyses address redo after conventional TESE, a materially more favourable baseline than redo after a failed micro-TESE, and their retrieval rates are therefore not directly transferable to the redo micro-TESE setting. More recently, Tai et al. [28] explored the feasibility of repeating micro-TESE within six months in selected patients, suggesting that a rigid waiting period may not be necessary in every case, although this observation rests on a small, selected cohort.

A further dataset speaks directly to the effect of repetition itself. Amer et al. [29] analysed a prospectively maintained database of 330 men with NOA at a single specialised centre, divided equally into first, second and third timers. Several of their findings run against the conventional prognostic account: a higher preoperative FSH was associated with a favourable outcome among first-timers (*p* = 0.015), Sertoli cell-only syndrome (SCOS) on histology was likewise associated with a favourable first-attempt outcome (*p* = 0.001), and among second-timers older—rather than younger—age predicted successful retrieval (*p* = 0.031). The authors concluded that spermatozoa may be recovered despite a high FSH and that preoperative histopathology is not indispensable in selecting men for a further attempt. These results warrant caution, since the three groups consisted of different patients rather than the same patients followed over time, and the direction of several associations is difficult to reconcile with the larger series discussed above. They nonetheless reinforce the central practical point that no single preoperative variable should be used on its own to exclude a man from a redo procedure.

Comparisons with non-NOA populations provide useful context. In a series of 439 testicular sperm aspiration procedures—366 primary and 73 redo—Alahmari et al. [30] reported a retrieval rate of 99% in men with obstructive azoospermia, significantly higher than in men with severe oligoasthenoteratozoospermia, with repeat aspiration from the same testicular unit remaining highly productive in the obstructive group. Where spermatogenesis is intact and the difficulty is one of access, repeating a retrieval procedure carries almost no prognostic penalty. The low success rate of redo micro-TESE in NOA is therefore more likely due to limited remaining sperm production than to the procedure being performed a second time. This is important when counselling couples who may have heard that second attempts are often successful in other types of azoospermia.

The unusually high success rate reported by Yücel et al. [22] deserves particular attention, as it may give patients an overly optimistic view of the chances of successful sperm retrieval. In that retrospective series, 49 men underwent a second micro-TESE after a failed first procedure and 21 (42.8%) had successful retrieval. Both procedures were performed at the same institution, by the same team, using an identical protocol and without hormonal preparation—a combination that has been interpreted as evidence that operator experience and procedural consistency may be decisive in redo outcomes [31]. Consistent with that interpretation, four men in the cohort had normal spermatogenesis on the histology obtained at the failed first procedure, and all four had spermatozoa retrieved at the redo attempt [3]. Where an initial micro-TESE has been performed elsewhere, or where its technical adequacy is uncertain, this observation is arguably of greater practical importance than any pharmacological adjunct.

Spahovic et al. [4] reported a smaller single-centre retrospective experience. Among 33 men with NOA and a negative first micro-TESE, histopathology showed SCOS in 14 (43%), a mixed pattern in seven (21%), germ cell maturation arrest in five (15%), seminiferous tubule hyalinization in five (15%), and hypospermatogenesis in two (6%); spermatogenic cells were identified in only three of the 33 specimens (9.1%). On the basis of these findings, a “second-look” procedure—preceded by three months of gonadotropin stimulation—was offered to the three eligible patients, those with hypospermatogenesis alone or a mixed hypospermatogenesis/SCOS pattern, and yielded sperm in one. Across the whole cohort, mean FSH was significantly lower in men in whom germ cells were identified histologically than in those without (17.26 ± 3.11 vs. 24.28 ± 4.71 IU/L, *p* = 0.038), whereas testosterone, LH, and prolactin did not differ significantly between groups. In an earlier series, Haimov-Kochman et al. [32] found that repeat TESE after a negative first attempt was successful in roughly one-third of patients—a figure at the upper end of the reported range, which most plausibly reflects favourable patient selection.

**Figure 2:**
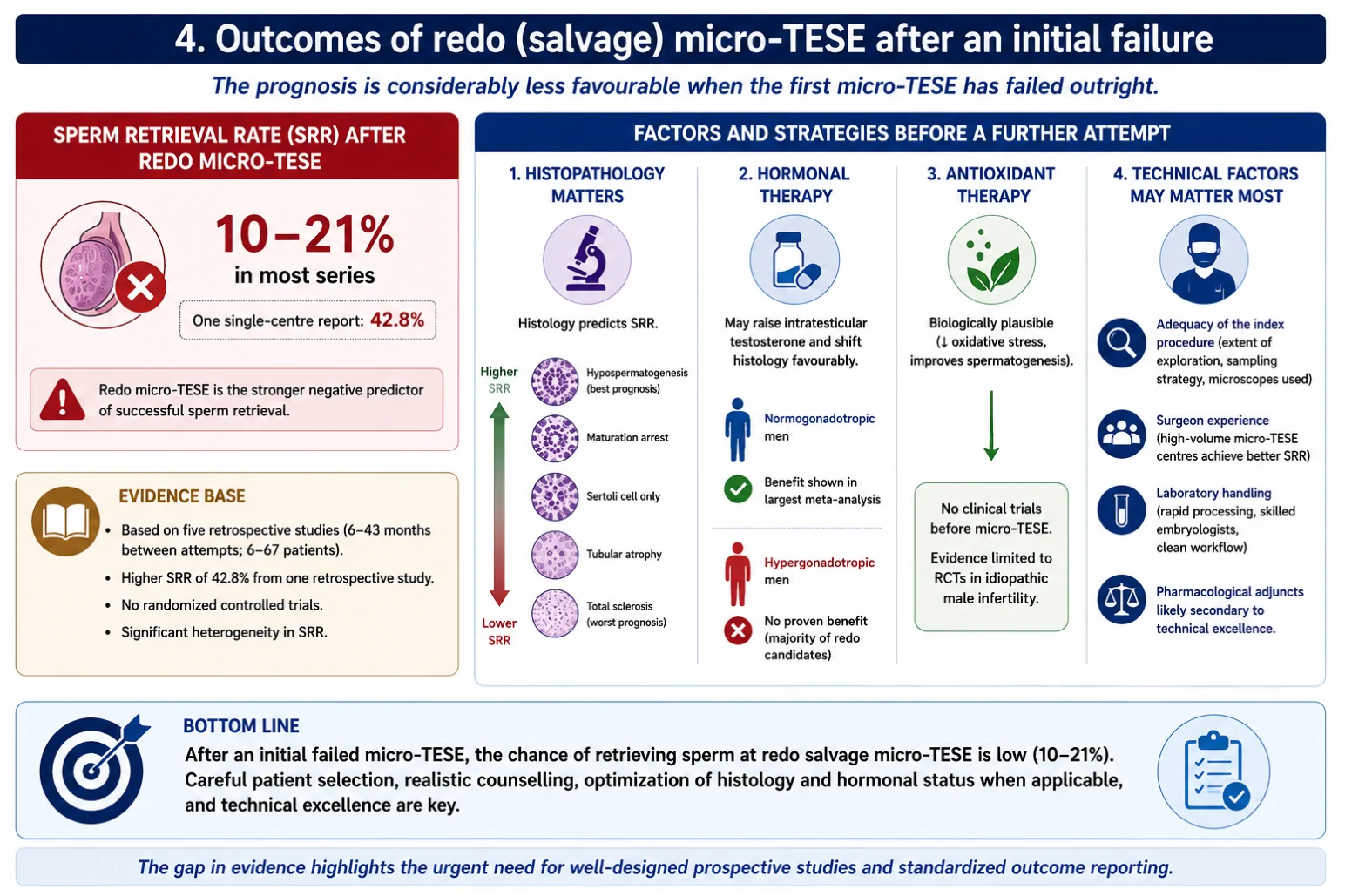
Redo micro-TESE after an initial failure. The figure summarizes the less favourable retrieval prognosis and the factors considered before another attempt. Outside genetically selected Klinefelter syndrome cohorts, most series report SRRs of 10–21%, with one single-centre series reporting 42.8% [3,22]. Histopathology may inform prognosis, but sampling error limits prediction. Hormonal treatment may increase intratesticular testosterone and alter histology; subgroup findings do not establish differential efficacy by hormonal status or benefit specifically before redo micro-TESE. Antioxidants remain untested in this setting. Assessment of the adequacy of the index operation, surgeon experience, and laboratory tissue processing should inform counselling alongside the uncertain benefits of pharmacological adjuncts. The evidence is heterogeneous and predominantly retrospective, and no randomized trial establishes an optimal redo strategy. ICSI, intracytoplasmic sperm injection; micro-TESE, microdissection testicular sperm extraction; RCT, randomized controlled trial; SRR, sperm retrieval rate.

Consistent with this overall pattern, Alriyalat et al. [2], in a retrospective analysis of 152 men with NOA, found that first-time micro-TESE achieved sperm retrieval in 64.6% of cases compared with 28.8% among the 73 patients (48%) who had previously undergone at least one testicular sperm extraction elsewhere (*p* < 0.01). The study does not report whether those prior procedures had been successful or unsuccessful, so this “repeated” group is a mixed population rather than a uniform redo cohort; nonetheless, it illustrates the same overall gradient described by Elbardisi [3] and Zhang [26] and their colleagues. Men undergoing repeat procedures were significantly older, had a higher smoking prevalence, and had a less favourable hormonal profile (higher FSH and LH and lower total testosterone) than first-time patients. Within the repeat subgroup itself, however, age, smoking, and hormone levels did not differ significantly between those with a positive and a negative outcome. KS was the only factor significantly associated with outcome in that subgroup, being more prevalent among patients with a negative repeat result (14/52, 27%) than a positive one (1/21, 5%; *p* = 0.031). The prior retrieval outcomes were not reported, preventing attribution of this association to previous failure. For context, Corona et al. reported an overall pooled SRR per TESE cycle of 44% (95% CI: 39–48%) across 37 studies involving 1248 men with KS [33]. This estimate includes conventional and microdissection TESE and is not a redo-specific prognosis (Figure 2).

## 5. Predictors of Success in Repeat and Redo Micro-TESE

No single variable reliably predicts the outcome of a further micro-TESE. The factors most often examined are summarized in Table 1 and discussed in turn below. The difficulty is not one of insufficient candidate variables. As Kaltsas et al. [34] set out in a recent appraisal of the field, conventional clinical, endocrine and histological parameters continue to perform poorly as stand-alone predictors of retrieval, and attention has shifted toward non-invasive molecular markers and, more speculatively, toward machine-learning models that combine several weak predictors—approaches that remain unvalidated at scale and, so far, entirely untested in men presenting for a second procedure.

### 5.1. Outcome of the Prior Attempt

Where the preceding procedure was successful, its outcome carries the largest effect size of any predictor identified in this review, and it is for that reason considered first. This “success breeds success” pattern was quantified by Ghalayini et al. [13], with odds ratios of 10.1, 5.6, and 16.5 at the second, third, and fourth attempts, respectively, and is broadly consistent with the clinical experience reflected in other reviewed studies, including the series of Vernaeve et al. [18] and Kamal et al. [16].

### 5.2. Testicular Histopathology

Testicular histology is the most consistently reported predictor of successful sperm retrieval, whether at a first, repeat, or redo procedure. Hypospermatogenesis, the least severe pattern, is associated with the highest retrieval rates—reported at 73–100% across first-attempt series [1] and 94.8% in the largest repeat series reviewed here [13]—whereas SCOS, representing complete germ cell aplasia, is associated with the lowest (approximately 22–41%); maturation arrest occupies an intermediate position [1]. Elbardisi et al. [3] note, however, that comparison across studies is hampered by the use of different classification and scoring systems—including the McLachlan et al. [42] classification, the Johnsen score [43], and Levin’s classification [44]—and by inconsistent definitions of “hypospermatogenesis.” In one included series, for example, the stated use of the McLachlan classification was contradicted by the reported absence of late spermatids in all 12 cases labelled hypospermatogenesis. These differences complicate pooling of results and limit the precision with which histology alone can be used to counsel patients before a redo attempt.

Two further observations qualify the histological account in the redo setting specifically. First, Amer et al. [29] found SCOS on index histology to be associated with, rather than against, successful retrieval in their first-timer group and concluded that preoperative histopathology was not essential to prediction. Second, in the KS-specific redo series of Salvarci and Balasar [35], every patient in whom spermatozoa were recovered at a second micro-TESE had SCOS on the pathology obtained at the failed first procedure, leading those authors to state explicitly that prior pathology offered no guidance for the redo decision. Set against these are data from large first-attempt cohorts in which quantitative scoring performs strongly: in 600 men with NOA, Mohamed et al. [40] found the Johnsen score to be the single most powerful independent predictor of retrieval on logistic regression (OR: 1.11, 95% CI: 1.09–1.12, *p* < 0.001), with an area under the ROC curve of 0.98 at a threshold of 1.5. The apparent contradiction is mainly explained by differences in the patient population. Histology is useful for predicting sperm retrieval before a first micro-TESE. However, among men who have already had an unsuccessful micro-TESE, most tend to have unfavourable histology, making it less useful for predicting the outcome of another attempt. In the Mohamed cohort, the Johnsen score was obtained during the first procedure and was therefore a postoperative finding rather than a preoperative predictor. This is an important limitation of using histology to guide decisions about repeat micro-TESE.

A more fundamental limitation is sampling error. Kaltsas et al. [36] compared diagnostic and therapeutic testicular biopsy in 50 men with NOA undergoing micro-TESE and found that diagnostic biopsy had failed to demonstrate spermatozoa in half of the men in whom the therapeutic procedure subsequently succeeded; no preoperative variable they examined—age, FSH, LH, total testosterone, prolactin, oestradiol, or systemic inflammatory indices—reliably predicted the outcome. Because spermatogenesis in NOA is focal, a biopsy reports the tubules that happened to be sampled rather than the state of the testis, and a negative index histology is therefore weaker evidence against a further attempt than its prominence in the predictive literature would suggest. This is the strongest single argument for treating unfavourable histology as a factor that lowers, rather than extinguishes, the probability of success at a redo procedure.

### 5.3. Serum FSH and Emerging Biomarkers

Elevated FSH is repeatedly, though not universally, linked to lower odds of sperm retrieval. In the redo literature synthesized by Elbardisi et al. [3], the clearest evidence comes from Yücel et al. [22], in whom a lower preoperative FSH was significantly associated with retrieval at redo micro-TESE (20.4 ± 9.7 vs. 31.2 ± 10.4 mIU/mL, *p* = 0.013) and remained an independent predictor in a multivariable model including age, FSH, LH, and Johnsen score (*p* = 0.032). The comparable finding in the series of Spahovic et al. [4] relates to the presence of germ cells on histology rather than to the outcome of the redo procedure itself and should not be read as a second independent confirmation. Ghalayini et al. [13] likewise found lower FSH to be significantly associated with success at the first micro-TESE attempt, although this association did not persist at subsequent attempts. Conversely, Ramasamy et al. [37] reported that high serum FSH did not compromise the success of micro-TESE in men with NOA—SRRs were 51% below 15 IU/mL, 60% at 15–30 IU/mL, 67% at 31–45 IU/mL, and 60% above 45 IU/mL—and other studies report no significant FSH–SRR relationship, particularly for TESE performed after a prior negative biopsy. FSH should therefore be regarded as a helpful but imperfect adjunct to histology rather than a stand-alone gatekeeping test. More recently, interest has grown in combining FSH with other reproductive hormones: a systematic review and meta-analysis by Pozzi et al. [38] evaluated FSH, inhibin B, and anti-Müllerian hormone as predictors of retrieval at micro-TESE and concluded that, while each marker carries some prognostic information, neither a single marker nor a combination of markers yet provides sufficient discriminatory power to exclude patients from a repeat or redo attempt.

Two more recent datasets extend this picture in opposite directions. Amer et al. [29] found that higher preoperative FSH was associated with successful sperm retrieval at the first attempt. They concluded that high FSH alone should not exclude a patient from treatment, consistent with Ramasamy et al. [37] and arguing against the use of a specific FSH cut-off for patient selection. Conversely, in a large first-attempt cohort, Mohamed et al. [40] found not only lower FSH (13.2 ± 11.3 vs. 21.7 ± 17.5 mIU/mL) and LH (8.1 ± 4.6 vs. 11.9 ± 10.3 mIU/mL) but also lower oestradiol (34.7 ± 16.9 vs. 70.9 ± 51.3 pg/mL) and prolactin (10.4 ± 4.9 vs. 17.2 ± 12.0 ng/mL) among men with successful retrieval (all *p* ≤ 0.006) and identified the hormonal profile together with testicular volume as independent predictors on multivariable analysis. The discriminatory performance of the individual endocrine markers in that study was nonetheless modest—the reported areas under the curve for oestradiol and prolactin were 0.22 and 0.34 respectively—and the finding has not been reproduced in a redo population.

Interest has also turned to seminal rather than serum markers. Amer et al. [39] measured seminal plasma leptin and resistin in 53 men with NOA—27 undergoing a first micro-TESE and 26 a repeat procedure—alongside 28 normozoospermic controls. Seminal leptin was the only variable that correlated significantly with retrieval, and a threshold of 4.05 ng/mL predicted success among first-time patients with a sensitivity of 73.3% and a specificity of 75% (AUC: 0.747, *p* = 0.030); resistin discriminated poorly (AUC: 0.678, *p* = 0.118). The result of greatest relevance here, however, is the negative one: in the repeat micro-TESE subgroup, neither marker predicted outcome (AUC: 0.633, *p* = 0.362, for leptin; AUC: 0.371, *p* = 0.380, for resistin). This is a compact illustration of a pattern that recurs throughout the present review—that predictors validated in first-attempt populations frequently lose their discriminatory power once the population has been filtered by a prior failure—and it argues that candidate biomarkers must be evaluated in redo cohorts specifically before being used to counsel these men.

### 5.4. Testicular Volume

Larger testicular volume was significantly associated with successful retrieval at the first and second attempts in the Ghalayini cohort [13], but not at the third or fourth. Conversely, in the Özman et al. [41] cohort, men with successful redo retrieval had smaller mean testicular volumes than those without (8.2 ± 5.4 vs. 11.3 ± 5.3 mL, *p* = 0.01). This unexpected finding may be explained by the inclusion of KS patients in the successful group: seven out of the 23 successful retrievals (30%) occurred in the 14 men with KS, a condition characterised by small, fibrotic testes in which isolated foci of spermatogenesis may nonetheless persist. Testicular volume alone therefore neither reliably predicts nor precludes successful retrieval and must be interpreted alongside histology and genetics—a conclusion consistent with the predictive modelling of Ramasamy et al. [45]. In the largest recent first-attempt series, Mohamed et al. [40] again found significantly larger testicular volumes among men with successful retrieval (8.7 ± 2.8 vs. 6.8 ± 3.0 mL, *p* < 0.001), which is consistent with the direction of effect reported at the first and second attempts but does not resolve the discrepancy observed at redo.

### 5.5. Interval Between Procedures

A minimum recovery interval of approximately six months between attempts is widely recommended, reflecting the time required for the testis to recover from the physiological and microvascular effects of the prior biopsy; a period of 6–12 months is advised by several authors [1,4,11]. Ghalayini et al. [13] provide the clearest quantitative support: each additional month of interval increased the odds of success at the second attempt by 1.3-fold, although this effect was no longer significant beyond the second attempt. Ramasamy and Schlegel [46] similarly examined the effect of a prior biopsy on subsequent retrieval and reinforced the importance of adequate recovery time. More recent work by Tai et al. [28], however, suggests that an interval of under six months may still be feasible in carefully selected patients, indicating that the optimal interval may ultimately be individualized rather than fixed. Non-surgical considerations—in particular, female partner age, ovarian reserve, and assisted reproduction scheduling—may legitimately modify the decision [11].

### 5.6. Aetiological Subgroups: Klinefelter Syndrome, Cryptorchidism, Mumps Orchitis and Y-Chromosome Microdeletions

Findings regarding KS are inconsistent across the reviewed literature. In the redo series summarized by Elbardisi et al. [3], second-attempt retrieval in KS patients ranged widely, from seven out of 14 (50%) in the Özman cohort [41] to three out of 18 (16.7%) in the KS-only cohort of Haliloglu et al. [23]. By contrast, in the Alriyalat et al. [2] cohort, KS was significantly more common among patients with a failed repeat procedure. This discrepancy most likely reflects differences in patient selection, prior hormonal optimization, and the extent of residual focal spermatogenesis rather than a genuinely contradictory biological effect, and argues for individualized counselling of KS patients regarding redo attempts rather than a uniform recommendation—consistent with the broader systematic review of KS outcomes by Corona et al. [33]. Evidence on Y-chromosome microdeletions derives from first-attempt populations and is extrapolated to the redo setting: complete AZFa or AZFb deletions are essentially incompatible with successful retrieval, whereas AZFc deletions, the most common microdeletions, are compatible with retrieval rates of approximately 50–71% [1,5]. These genotype-specific findings, including the statement on complete AZFa/AZFb deletions, are not based on dedicated redo cohorts; the quoted AZFc retrieval rates should not be presented as redo-specific prognostic estimates. Genetic screening should therefore precede any planned redo attempt.

A dedicated redo series adds usefully to the KS picture. Salvarci and Balasar [35] reviewed 71 men with non-mosaic KS managed at a single centre over a decade, of whom 35 underwent a second micro-TESE. Spermatozoa were recovered in 15 out of 35 (42.8%) overall, but the rate differed sharply according to the outcome of the index procedure: nine out of 15 (60%) where the first attempt had succeeded, against six out of 20 (30%) where it had failed—the same “success breeds success” pattern was reproduced within a genetically defined population. Among the six men who converted from a negative first to a positive second procedure, two pregnancies (33%) followed and one resulted in a live birth. None of the variables—FSH, LH, testosterone, or testicular volume—distinguished successful from unsuccessful redo attempts. Two practical observations from that series deserve emphasis: operating time in previously negative patients was substantially longer than in men with normally sized testes, which the authors attributed to diffuse fibrosis left by the index operation, and no preoperative variable—prior histology included—was found that would have guided case selection. Read alongside the Özman [41] and Haliloglu [23] data, the defensible position is that a redo attempt in KS is neither futile nor routinely indicated and that the outcome of the first procedure remains the most informative single item in the counselling discussion.

Other aetiologies also require separate consideration. In men with previously corrected undescended testes (cryptorchidism), Özman et al. reported sperm retrieval in 2/11 (18.2%) after a failed first micro-TESE [41]. By contrast, Gao et al. reported retrieval in 17/21 men (81.0%) with cryptorchidism and 9/12 (75.0%) with mumps orchitis in a general micro-TESE cohort, without separate repeat/redo outcomes; these rates therefore cannot be used to predict success after prior failure [47]. Partial AZFa, AZFb and AZFb+c deletions should not be equated with complete deletions. Partial AZFa deletions affecting USP9Y have variable phenotypes, whereas selected partial AZFb and atypical AZFb+c deletions may retain spermatogenesis; precise deletion-extension analysis is required for interpretation [48]. Aoki et al. described successful micro-TESE in a cryptozoospermic man with partial AZFb deletion, but no pregnancy followed two embryo transfers, and this was not a redo case [49]. The Özman redo cohort included one man with partial AZFb deletion, but a separately attributable outcome could not be established from the available report [41]. Thus, direct redo evidence is limited for corrected cryptorchidism, and reliable subgroup-specific repeat/redo estimates remain unavailable for mumps orchitis and these partial deletions.

## 6. Medical Optimization Before a Repeat or Redo Procedure

A second—or third, or fourth—testicular operation entails potential structural and endocrine harm, alongside financial and psychological costs. Although not specific to repeat surgery, a systematic review and meta-analysis found mean serum testosterone declines at 6 months after TESE of 2.7 nmol/L in men with NOA and 4.1 nmol/L in men with KS, with recovery to baseline at approximately 18 and 26 months, respectively [50]. In one cohort included in that review, a testicular volume reduction exceeding 2 mL at 6 months occurred in 10/40 men (25%) after c-TESE and 2/80 (2.5%) after micro-TESE; these mixed azoospermia populations do not establish the incremental risk of a further procedure [50]. For repeat surgery specifically, Salvarci and Balasar attributed prolonged operating time in previously negative KS patients to diffuse fibrosis after the first operation [35], reinforcing the need to discuss potential tissue injury and endocrine impairment alongside the expected retrieval benefit. There has therefore been sustained clinical interest in medical strategies that might improve the odds of success beforehand, so that a further operation is undertaken only once the probability of benefit has been maximized. Two strategies dominate the literature and clinical practice: hormonal therapy, aimed at raising intratesticular testosterone and stimulating residual spermatogenic foci, and antioxidant therapy, aimed at counteracting oxidative stress. Both are discussed below, followed by shorter discussions of varicocelectomy and of non-pharmacological strategies. Table 2 summarizes the strategies proposed before a further attempt and the evidence supporting each.

### 6.1. Hormonal Therapy Before Repeat or Redo Micro-TESE

#### 6.1.1. Rationale

Intratesticular testosterone (ITT) concentrations far exceed serum levels and are essential for the later stages of spermiogenesis, yet serum testosterone correlates poorly with ITT, so that a normal serum testosterone does not exclude a functionally low local androgen environment [51]. This observation underlies the rationale for hormonal stimulation in NOA: by raising ITT through gonadotropin administration, or by manipulating the hypothalamic–pituitary–gonadal axis with aromatase inhibitors or selective oestrogen receptor modulators (SERMs), clinicians have sought to rescue foci of arrested spermatogenesis and thereby improve the odds of retrieval at a subsequent procedure [2]. Interest in such adjuncts is greatest before a redo attempt, when the baseline probability of success is lowest. The mechanistic case has been set out in detail by Shiraishi [52], who argued that the long-standing assumption that hormonal treatment must be futile in the presence of elevated gonadotropins is not well founded: serum and intratesticular androgen environments are dissociated, the androgen receptor on Sertoli cells is the principal effector of androgen action on spermatogenesis, and hCG-based regimens demonstrably raise ITT. That review also noted that a small number of men with NOA develop spermatozoa in the ejaculate under such treatment—an outcome that, where it occurs, obviates a further operation altogether and is arguably the most valuable endpoint hormonal therapy could deliver in this setting.

#### 6.1.2. Gonadotropin-Based Regimens

The most extensively studied regimens before redo micro-TESE use human chorionic gonadotropin (hCG), which mimics luteinizing hormone and stimulates Leydig cell testosterone production, often combined with recombinant FSH (rhFSH) to support Sertoli cell function directly. Shiraishi et al. [53] treated men with NOA using hCG 5000 IU subcutaneously three times weekly for three months; hCG was continued for a further one to two months if plasma FSH remained high, and rhFSH 150 IU three times weekly was added for two months if FSH fell below 3 mIU/L under hCG-induced negative feedback. They reported sperm retrieval in six out of 28 treated patients (21%) at subsequent micro-TESE compared with zero out of 20 untreated patients, in a prospective study that lacked a randomized control arm. In a later multi-institutional prospective study using hCG followed by rhFSH, the same group [24] reported a more modest redo retrieval rate of two out of 21 (10%), with one resulting live birth.

A related mechanistic study by Shinjo et al. [54], using an almost identical hCG-based protocol, found that ITT increased significantly and consistently after treatment (from 273.6 ± 134.4 to 1348.1 ± 505.4 ng/mL, *p* < 0.0001) and that hCG-based therapy promoted spermatogonial proliferating cell nuclear antigen (PCNA) expression, particularly in men from whom spermatozoa were ultimately obtained at the second micro-TESE. Histologically, responders showed a shift from maturation arrest toward hypospermatogenesis, whereas non-responders showed basement membrane thickening and interstitial fibrosis. These findings offer biological plausibility for the approach even though the associated retrieval rate (three out of 20, 15%) remained modest.

Amer et al. [55] tested a variant protocol combining one month of testosterone-mediated downregulation of the hypothalamic–pituitary axis with three subsequent months of hCG and FSH, reporting sperm retrieval in two out of 20 treated patients (10%) versus zero out of 20 controls—a difference that did not reach statistical significance (reported *p* = 0.072). It was not clear from the study design whether all cases and controls had undergone a conventional TESE or a micro-TESE at their first procedure [3]. Spahovic et al. [4] used a comparable gonadotropin regimen for three months in the three patients selected for second-look micro-TESE, one of whom achieved retrieval.

Recombinant gonadotropin therapy has also been explored as a proof-of-concept strategy rather than as hormonal therapy before redo micro-TESE specifically. Laursen et al. [56] treated men with NOA using recombinant gonadotropins to determine whether spermatogenesis could be meaningfully stimulated, adding mechanistic support to the broader hormonal-therapy literature without the scale required to alter practice.

#### 6.1.3. Aromatase Inhibitors and Selective Oestrogen Receptor Modulators

An alternative or complementary approach uses aromatase inhibitors such as anastrozole, or SERMs such as clomiphene citrate, to reduce oestrogenic negative feedback on the hypothalamic–pituitary axis and thereby raise endogenous LH, FSH, and testosterone. In the largest redo-specific cohort using this approach, Özman et al. [41] reported outcomes in 125 men undergoing redo micro-TESE after a failed first procedure, of whom 42 received hormonal optimization—anastrozole where total testosterone was below 300 ng/dL or the testosterone/oestradiol ratio was below 10, or clomiphene citrate where LH was within the normal range, with weekly hCG added for those with an inadequate response. The overall SRR at redo was 18.4% (23/125), with subgroup rates of 17.4% (12/69) in idiopathic NOA and 50% (7/14) in KS. Critically, the difference in SRR between treated (11/42, 26.2%) and untreated patients (12/83, 14.5%) was not statistically significant (*p* = 0.11). That result, together with the absence of a randomized design, tempers enthusiasm for the approach.

#### 6.1.4. Exogenous FSH Monotherapy

Exogenous FSH administration in men with normal baseline FSH has been studied outside the redo setting. In a controlled study of 108 men with NOA and normal FSH, Aydos et al. [57] compared FSH injections three times weekly for three months with no treatment before micro-TESE and found a significantly higher retrieval rate in the treatment group (64% vs. 33%). The normal-FSH eligibility criterion limits generalizability; this study does not establish that baseline hormonal status modifies treatment efficacy (Section 6.1.5). The study nonetheless predates and is not specific to the redo setting, and the effect size has not been replicated.

#### 6.1.5. Pooled Evidence and Guideline Position

The individual studies above are small, heterogeneous in regimen, patient selection, and outcome definition, and mostly lack rigorous randomization or blinding—limitations emphasized by Elbardisi et al. [3], who concluded that the available evidence is insufficient to identify an optimal hormonal stimulation protocol, if any, before redo surgery. The most authoritative summary comes from the systematic review and meta-analysis by Tharakan et al. [51], which pooled 22 controlled studies totalling 1706 men with NOA. Across all included studies, hormonal therapy of any class was associated with a modestly higher SRR than placebo or no treatment (OR: 1.96, 95% CI: 1.08–3.56, *p* = 0.03), a result that persisted after exclusion of a study restricted to KS patients (OR: 1.90, 95% CI: 1.03–3.51, *p* = 0.04).

The normogonadotropic subgroup estimate was an OR of 2.13 (95% CI: 1.10–4.14; *p* = 0.02), whereas the hypergonadotropic estimate was an OR of 1.73 (95% CI: 0.44–6.77; *p* = 0.43) [51]. Significance in one subgroup and not the other does not demonstrate a treatment-by-hormonal-status interaction. These findings therefore cannot establish that benefit is confined to normogonadotropic men or absent in hypergonadotropic men. Bias and imprecision limit the evidence, and redo-specific efficacy remains uncertain. Empirical hormonal pretreatment should not be presented as established care; current EAU guidance advises against stimulation before TESE in NOA outside clinical trials [58].

### 6.2. Antioxidant Therapy Before Repeat or Redo Micro-TESE

#### 6.2.1. Biological Rationale

Oxidative stress, defined as an imbalance between reactive oxygen species (ROS) and the antioxidant capacity of seminal plasma and testicular tissue, is increasingly recognized as a contributor to impaired spermatogenesis and testicular dysfunction. Oxidative stress is characterized as a common final pathway in testicular dysfunction, implicating ROS-mediated damage to germ cells, Sertoli cells, and the blood–testis barrier across multiple causes of male infertility, including NOA [59]. Dutta et al. [60] subsequently reviewed the interplay between oxidative stress, testicular inflammatory pathways, and impaired male reproduction, noting that chronic local inflammation and ROS accumulation may perpetuate a cycle of germ cell apoptosis and fibrosis resembling that seen histologically in maturation arrest and SCOS. This mechanistic literature provides a coherent rationale for antioxidant supplementation—vitamin E, vitamin C, coenzyme Q10, N-acetylcysteine, L-carnitine, zinc, and selenium, alone or in combination—as a potential adjunct before repeat or redo micro-TESE, based on the premise that reducing oxidative burden might support residual spermatogenic foci.

#### 6.2.2. Evidence in General Male Subfertility

The strongest available evidence for antioxidant therapy comes not from the NOA or TESE literature but from the broader male subfertility literature, most authoritatively summarized in a series of Cochrane systematic reviews. Smits et al. [61] reviewed 61 randomized controlled trials involving 6264 subfertile men and comparing 18 oral antioxidants, finding that supplementation may increase live-birth rates (OR: 1.79, 95% CI: 1.20–2.67; seven trials, 750 men) and clinical pregnancy rates. The certainty of this evidence was rated low, owing to poor reporting of randomization methods, inconsistent reporting of live-birth and pregnancy outcomes, frequently unclear or high attrition, and imprecision arising from small trials with low event rates. The subsequent update by de Ligny et al. [61] reached similarly cautious conclusions, reaffirming a possible benefit for pregnancy and live birth while emphasizing that the overall quality of the evidence base remains low to very low and that large, well-designed, placebo-controlled trials reporting live birth are still required.

#### 6.2.3. Evidence Specific to NOA and TESE/Micro-TESE

Critically, the Cochrane evidence is drawn overwhelmingly from men with oligozoospermia or asthenozoospermia rather than azoospermia, and none of the redo micro-TESE studies reviewed here—including the nine series synthesized by Elbardisi et al. [3]—reported using or evaluating antioxidant therapy as a pre-procedure intervention. This gap is noted explicitly in the most recent narrative review of micro-TESE risks and benefits [11], which states that in the setting of redo micro-TESE after a failed first procedure, varicocelectomy, hormonal stimulation, antioxidants, and anti-inflammatory treatment should not be presented to patients as proven means of improving success and which separately concludes that antioxidants, anti-inflammatory approaches, and intraoperative or perioperative testosterone supplementation should not be recommended routinely in the absence of clinical evidence. This is a materially more cautious position than that taken in the general male subfertility literature, reflecting both the very small number of azoospermia-specific trials and mechanistic uncertainty as to whether a systemic reduction in oxidative stress can influence the highly localized, patchy foci of residual spermatogenesis that determine success in NOA.

#### 6.2.4. Interpreting the Evidence for Clinical Counselling

The antioxidant literature relevant to repeat or redo micro-TESE can be summarized in three points. First, the biological rationale is sound and well described [59,60]: oxidative stress plausibly contributes to the testicular dysfunction underlying NOA and could in principle be a modifiable target. Second, the general male-subfertility evidence base [61] offers low-certainty but not negligible support for benefit in terms of pregnancy and live-birth outcomes, at minimal cost and low risk of harm, which explains the intervention’s continued empirical use. Third, and most important for counselling before repeat or redo micro-TESE, no adequately powered, controlled, NOA- or micro-TESE-specific trial has demonstrated that antioxidant supplementation improves surgical retrieval rates, and recent expert reviews caution explicitly against presenting antioxidants as a proven pre-procedure strategy in this setting [11]. Clinicians may reasonably discuss antioxidant supplementation as a low-risk, biologically plausible adjunct supported by the general subfertility literature, provided they are transparent that its specific value before a repeat or redo micro-TESE remains unproven and represents an important target for prospective research.

### 6.3. Varicocelectomy Before a Repeat or Redo Attempt

Varicoceles are present in approximately 5% of men with NOA, and their role in the pathophysiology of azoospermia—coincidental or contributory—remains debated [3,6]. Only two out of the nine redo series reviewed by Elbardisi et al. [3] examined the impact of varicoceles on retrieval, and neither demonstrated a clear benefit from prior repair. In the cohort of Yücel et al. [22], clinical grade 1–2 varicoceles were present in four men in the positive and five in the negative retrieval group, but patients who had undergone varicocelectomy were not distinguished from those who had not. In the cohort of Özman et al. [41], 13 men had undergone varicocelectomy at least three months before their first micro-TESE and none achieved sperm retrieval at the redo procedure, whereas one out of 12 men with an untreated palpable varicocele—a grade 3 lesion—did. Neither study evaluated men who underwent varicocelectomy specifically in the interval between a failed first procedure and a redo attempt, which is the comparison of interest.

This contrasts with more favourable data from the broader NOA literature. A meta-analysis by Esteves et al. [62], pooling 11 retrospective and seven prospective studies comprising 468 men with NOA and clinical varicoceles, reported an SRR of 61% after varicocelectomy versus 39% without (OR: 2.7, 95% CI: 1.69–4.14), suggesting that varicocele repair may have a role in selected NOA patients being considered for a first micro-TESE, particularly those with hypospermatogenesis or late maturation arrest on histology [1]. Its specific value before a redo procedure, however, remains unproven, and the most recent expert review again cautions against presenting varicocelectomy as a proven means of improving redo micro-TESE success [11].

A broader rationale for considering repair in this population is set out in the review by Kaltsas et al. [63], which notes that varicoceles are found in roughly 4–14% of men with NOA and that repair may return spermatozoa to the ejaculate, may raise the retrieval rate at subsequent surgical extraction, and may improve ICSI pregnancy rates even where no ejaculated sperm appear—while spontaneous pregnancy remains uncommon. That review also draws attention to a non-fertility endpoint of some relevance here: varicocelectomy may improve Leydig cell secretory function and endogenous androgen production. Given that cumulative androgen loss is one of the principal costs of repeated micro-TESE, this is an argument for considering repair on its own merits in a man with a clinical varicocele, independently of whether it improves the odds at a further retrieval.

### 6.4. Non-Pharmacological Strategies: Combined Single-Session Retrieval and FNA-Guided Redo

Rather than relying on medical pre-treatment before a delayed second procedure, two technical strategies aim to maximize yield without a repeat operation, or to make a subsequent redo procedure more efficient and targeted.

Barda et al. [64] reported a retrospective within-subject cohort of 93 men with NOA who underwent both micro-TESE and trifocal conventional TESE sequentially within a single operative session, with specimens processed and analysed separately. The combined approach achieved a significantly higher retrieval rate (59/93, 63.4%) than either micro-TESE (58.1%, *p* = 0.041) or trifocal TESE (54.8%, *p* = 0.002) analysed alone, with sperm found exclusively in micro-TESE specimens in seven patients (7.5%) and exclusively in trifocal specimens in three (3.2%). Quantitative sperm yield was also higher with the combined approach (9.9 ± 5.1 sperm units) than with micro-TESE (3.9 ± 3.2) or trifocal TESE (6.0 ± 3.4) alone (*p* < 0.001), and among men with successful retrieval, those in whom both techniques yielded sperm had substantially more cryopreserved vials than those in whom only one did (12.4 ± 2.4 vs. 5.9 ± 1.2, *p* < 0.001). This dual-technique strategy follows earlier work by Marconi et al. [65], who proposed that combining trifocal and microsurgical extraction offers the highest yield in men with a low a priori chance of success, and it represents a plausible means of reducing the number of patients who would otherwise require a subsequent redo procedure. It has not, however, been compared directly with staged repeat micro-TESE in a randomized design.

A related consideration concerns the extent of the index dissection itself. Almajed et al. [66] reported a retrospective series of 103 men with NOA or cryptozoospermia undergoing a modified micro-TESE through a mini tunica albuginea incision with superficial tissue dissection, a technique designed to limit tissue injury while preserving retrieval yield. Its relevance to the present review is indirect but real and cuts both ways. Because the technical adequacy and the tissue cost of the index procedure both bear on what a subsequent attempt can achieve, a less destructive first operation may leave more retrievable tissue—and less of the fibrosis that lengthens and complicates redo surgery—whereas a deliberately limited first dissection may equally leave residual foci unexplored and thereby inflate the apparent value of a second look. No study has yet compared redo outcomes after standard versus tissue-sparing index procedures, and this is an obvious question for prospective work.

Testicular fine-needle aspiration (FNA) mapping samples multiple sites to locate residual spermatogenesis [67]. After failed micro-TESE, Jarvis et al. mapped 82 men: 24 were map-positive and 15 underwent guided retrieval, all successfully [25]. The 15/15 result was conditional on selection; documented retrieval occurred in 15/82 men who entered the mapping pathway, with outcomes unverified in those not operated on. Ten out of the 15 also received medical treatment or varicocele repair, preventing isolation of mapping’s contribution. These data establish neither comparative benefit nor a reliable negative predictive value. EAU guidance advises against prognostic FNA mapping before definitive TESE in NOA (weak recommendation) [58]. Mapping is therefore discussed as an investigational approach, not a routine redo strategy. Review of the prior operative, embryology and histology records remains appropriate before another attempt [11,68].

**Table 2:** Strategies proposed before a repeat or redo micro-TESE and the evidence supporting each.

Strategy	Rationale	Evidence in the Repeat or Redo Setting	Current Standing
hCG ± recombinant FSH	Raises intratesticular testosterone and supports Sertoli cell function	6/28 (21%) vs. 0/20 untreated [53]; 2/21 (10%) at redo [24]; 3/20 (15%), with rising intratesticular testosterone and a shift from maturation arrest to hypospermatogenesis in responders [54]; 2/20 (10%) vs. 0/20 after testosterone downregulation [55]	Biologically plausible; no study randomized or adequately powered, and none reached significance
Aromatase inhibitors or SERMs	Reduce oestrogenic negative feedback, raising endogenous LH, FSH, and testosterone	Largest redo cohort: 11/42 (26.2%) treated vs. 12/83 (14.5%) untreated, *p* = 0.11 [41]	Not significant; non-randomized allocation
Exogenous FSH monotherapy	Direct Sertoli cell stimulation in men with normal baseline FSH	64% vs. 33% before first micro-TESE [57]	Encouraging but outside the redo setting, unreplicated; the eligible population is normogonadotropic
Hormonal therapy (pooled)	—	Review: 22 studies, 1706 men; pooled OR: 1.96. Subgroups: normogonadotropic OR: 2.13; hypergonadotropic OR: 1.73 [51]	No demonstrated subgroup interaction; redo efficacy uncertain. Empirical stimulation before TESE should be restricted to trials [58]
Antioxidants	Oxidative stress contributes to impaired spermatogenesis [59,60]	None; no NOA- or micro-TESE-specific trial. Indirect support only from general male subfertility (live-birth OR: 1.79, low certainty) [61]	Unproven in this setting; expert reviews caution against routine use [11]
Varicocelectomy	Reduces a potentially reversible contributor to spermatogenic failure	0/13 after prior varicocelectomy at redo, vs. 1/12 with untreated grade 3 varicoceles [41]; varicocelectomy status not separated in the only other series [22]	Supported before a first micro-TESE (SRR: 61% vs. 39%, OR: 2.7) [62], but unproven before a redo
Combined single-session micro-TESE and trifocal TESE	Complementary sampling: targeted plus systematic	63.4% combined vs. 58.1% (*p* = 0.041) and 54.8% (*p* = 0.002) alone; higher sperm yield and more cryopreserved vials [64,65]	Promising; reduces the need for a further procedure, but not compared with staged repeat micro-TESE; tissue-sparing mini-incision variants may also alter what a later attempt can achieve [66]
FNA mapping before a redo	Samples multiple sites to locate residual spermatogenesis; adds a procedure before retrieval	24/82 map-positive; 15/15 operated men had retrieval (15/82 entering the pathway). Ten also had medical treatment or varicocele repair [25,67]	Investigational; negative predictive value unestablished. EAU advises against prognostic mapping before TESE (weak recommendation) [58]
Review of the index procedure; referral to a high-volume centre	Technical adequacy, operator experience, and andrology laboratory processing and search all influence redo yield; a negative micro-TESE records the failure of the dissection and of the laboratory search together [69]	Highest reported redo rate (42.8%) arose where both procedures were performed by one team using an identical protocol and no hormonal preparation [22,31]	No trial evidence, but plausibly of greater practical consequence than any pharmacological adjunct [11,68]

FNA, fine-needle aspiration; hCG, human chorionic gonadotropin; NOA, non-obstructive azoospermia; OR, odds ratio; SERM, selective oestrogen receptor modulator; SRR, sperm retrieval rate.

### 6.5. The Role of the In Vitro Fertilization Laboratory

Laboratory processing and sperm identification form part of the retrieval pathway, so review of the index embryology record can complement the operative assessment before a further attempt [69]. The reviewed studies do not quantify the independent effect of laboratory practice on redo success; its relevance to redo planning is therefore extrapolated from broader sperm-retrieval and ICSI evidence. Cryopreservation may reduce the need for further surgery when sufficient usable sperm are available: a meta-analysis of 17 studies and 1261 ICSI cycles in men with NOA found no statistically significant differences in fertilization, implantation or live birth between fresh and frozen–thawed testicular sperm [70]. These findings concern ICSI outcomes, not the efficacy of redo micro-TESE.

## 7. Interpreting the Evidence and Counselling the Patient

### 7.1. Interpretation for Clinicians and Research Priorities

**Figure 3:**
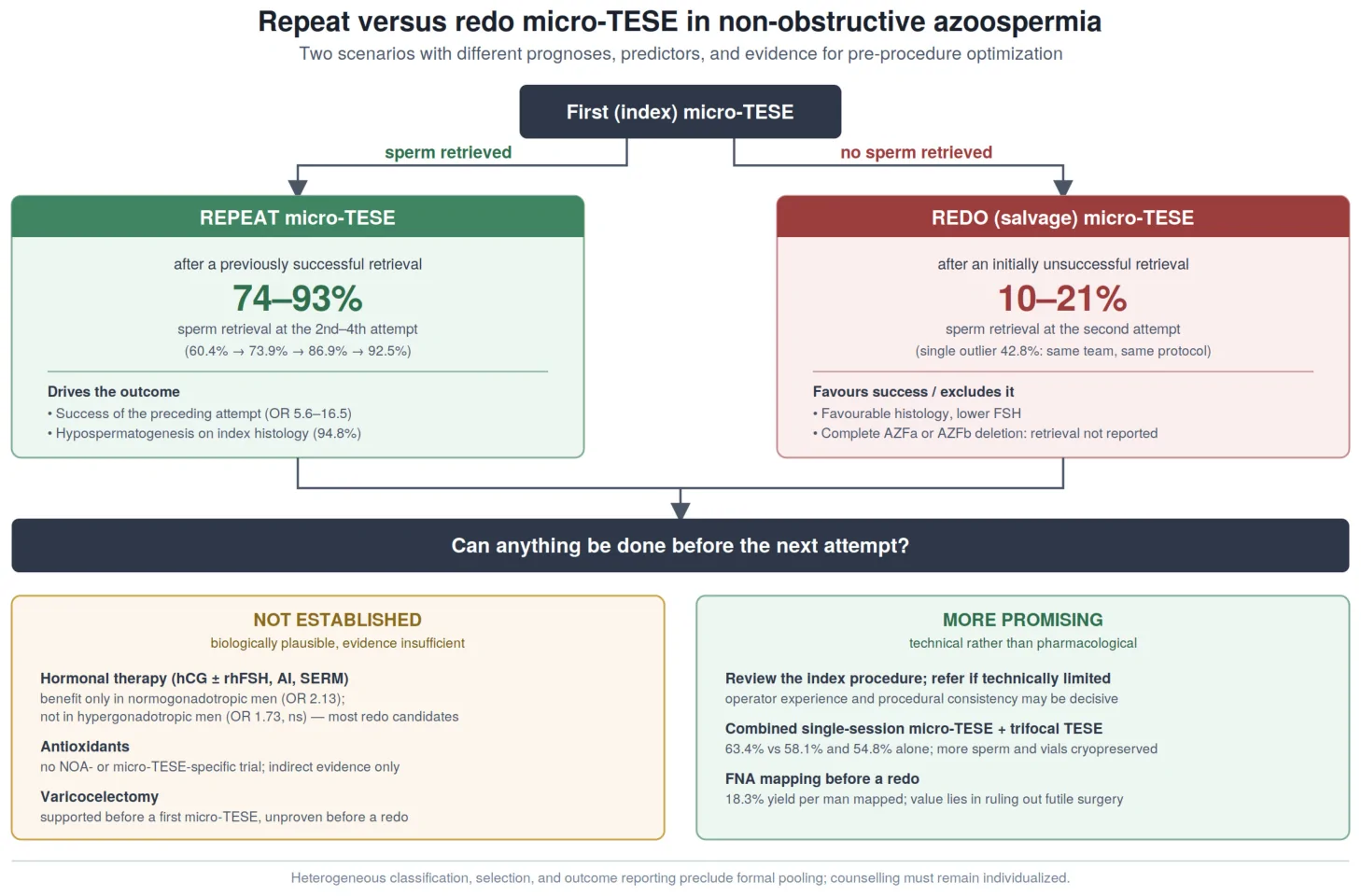
Repeat versus redo micro-TESE in non-obstructive azoospermia. The outcome of the index procedure separates two clinically distinct scenarios. Observed SRRs of approximately 74–93% at second to fourth attempts were reported in groups enriched for previous successful retrieval, with the preceding procedure outcome being the strongest determinant. The progressive increase reflects increasingly selected populations enriched for previous retrieval success and must not be quoted as the per-attempt prognosis for an unselected cohort. The apparent rise does not show that repeated surgery itself improves retrieval probability. Outside genetically selected Klinefelter syndrome cohorts, redo micro-TESE after an initially unsuccessful procedure yields spermatozoa in 10–21% of cases in most series; the single outlying report of 42.8% arose where both procedures were performed by one team using an identical protocol. Strategies proposed before a further attempt include medical pretreatment and technical approaches. Hormonal therapy, antioxidants and varicocelectomy lack established redo-specific efficacy; combined retrieval techniques require comparative validation. FNA mapping is investigational, and EAU guidance advises against its use as a prognostic procedure before definitive TESE [58]. AI, aromatase inhibitor; AZF, azoospermia factor; FNA, fine-needle aspiration; FSH, follicle-stimulating hormone; hCG, human chorionic gonadotropin; NOA, non-obstructive azoospermia; ns, not significant; OR, odds ratio; rhFSH, recombinant FSH; SERM, selective oestrogen receptor modulator; TESE, testicular sperm extraction.

Repeat micro-TESE after prior success and redo micro-TESE after failure represent distinct clinical populations (Figure 3). Higher retrieval rates at successive repeat attempts reflect selection for prior success and cannot be interpreted as improvement in the prognosis of an unselected cohort. Histopathology, endocrine status, testicular volume, interval and genetic findings should be interpreted together; inconsistent definitions and cohort selection limit their use as independent predictors [3,13,38,42,43,44].

Medical pretreatment deserves focused investigation in biologically defined NOA subgroups. Hormonal studies suggest possible benefit, but subgroup significance does not establish a difference in efficacy by hormonal status, and the small redo cohorts do not establish a causal treatment effect [51,53,54]. Antioxidants have a biological rationale, but evidence from general male subfertility cannot establish efficacy before repeat or redo micro-TESE [59,60,61]. Future prospective controlled trials should stratify patients by NOA aetiology, endocrine phenotype, index histology and prior retrieval outcome, and evaluate hormonal optimization and antioxidant therapy separately using predefined indications and regimens. Retrieval, live birth, adverse effects, treatment burden and cost should be measured. Biological plausibility and presumed affordability or tolerability should motivate such trials, not be treated as evidence of routine clinical benefit.

Technical review of the index procedure remains relevant to planning a further attempt. FNA-guided retrieval remains investigational and is not recommended for routine prognostic mapping before TESE [25,58,67]. Combined retrieval approaches require comparative evaluation, while evidence for varicocelectomy specifically before redo remains limited [62,64,65]. Observational differences between centres cannot establish whether surgical expertise, laboratory practice, patient selection or adjunctive treatment explains the outcome; standardized reporting is needed to separate these contributions [3,22,31].

Table 3 makes the principal sources of heterogeneity visible: small cohorts, differing genetic and histological case mixes, variable pretreatment, inconsistent interval reporting, and selective progression to another procedure. For example, a minimum surgical interval of 3 months [22] is not directly comparable with a mean of 2.2 years from surgery to FNA mapping [25]. Reproductive follow-up is sparse, and potential overlap among related reports further limits synthesis. These differences preclude a defensible pooled estimate and support prospective studies with transparent recruitment, standardized denominators and complete reproductive follow-up [3].

### 7.2. Information for Patient Counselling

Counselling should begin with what happened at the previous operation. Finding sperm previously makes another successful retrieval more likely but does not guarantee it. After a failed first micro-TESE, most published series report sperm retrieval in approximately 10–21% of men; these figures come from small, selected groups and are not an individual prediction [3]. Genetic findings, the previous tissue examination, and the surgical and laboratory records help frame the discussion. Finding sperm does not guarantee pregnancy or a live birth.

Patients should also understand the possibility of another unsuccessful operation, reduced testosterone, scarring, recovery time, and financial and emotional costs [35,50]. Hormonal treatment or antioxidants cannot currently be promised to improve redo success. Where pretreatment is considered for a specific clinical indication, the clinician should explain its purpose, uncertainty, potential adverse effects, monitoring, cost and any delay to surgery. The decision should reflect the couple’s circumstances, reproductive goals and preferences, including whether to proceed with further surgery or consider alternatives.

## 8. Conclusions

The outcome of the previous retrieval is central to planning another micro-TESE, and increasingly selected repeat cohorts should not be used to predict outcomes for unselected patients. Decisions require individualized assessment of the potential reproductive benefit and cumulative surgical burden. Hormonal optimization and antioxidant pretreatment are priorities for research in biologically selected NOA populations, but their routine use to improve repeat or redo outcomes is not established. Prospective controlled studies with standardized reporting and live-birth and safety outcomes are needed to guide practice.

## Figures and Tables

**Table 1:** Factors evaluated as predictors of successful sperm retrieval at repeat or redo micro-TESE.

Predictor	Association with Retrieval	Principal Evidence	Caveats
Preceding procedure outcome	Strongest effect identified; prior success predicts further success	Odds ratios of 10.1, 5.6, and 16.5 at the second, third, and fourth attempts [13]; 91.5% after a positive first procedure [16]; high cumulative recovery over up to six attempts [18]	Informative only in the repeat setting; provides no guidance after an initial failure
Index histopathology	Most consistently reported, though weakened by sampling error; hypospermatogenesis favourable, SCOS least favourable	94.8% vs. 40.9% (maturation arrest) vs. 31.5% (SCOS) at the first attempt [13]; 73–100% vs. 22–41% across first-attempt series [1]; better ICSI outcomes with hypospermatogenesis [21]	Classification systems differ (McLachlan, Johnsen, Levin) [3]; available only after the index procedure; differences lose significance by the third attempt; prior histology gave no guidance for the redo decision in a KS-specific series [35]; diagnostic biopsy missed spermatozoa in half of men whose therapeutic biopsy was positive [36]
Serum FSH	Lower FSH associated with success, but inconsistently	20.4 ± 9.7 vs. 31.2 ± 10.4 mIU/mL at redo, independent on multivariable analysis [22]; significant at the first attempt only [13]	Elevated FSH does not preclude retrieval (SRR: 51–67% across FSH strata) [37]; a high FSH has even been associated with favourable first-attempt retrieval [29]; no validated cut-off; an adjunct to histology, not a gatekeeping test
Inhibin B, AMH, seminal biomarkers	Some prognostic information	Systematic review and meta-analysis with FSH [38]; seminal plasma leptin ≥ 4.05 ng/mL predicted retrieval at first micro-TESE (AUC: 0.747) [39]; lower oestradiol and prolactin in successful first attempts [40]	Insufficient discriminatory power, alone or combined, to exclude patients from a further attempt; seminal leptin lost all predictive value in the repeat subgroup (AUC: 0.633, *p* = 0.362) [39]
Testicular volume	Direction of effect conflicting	Larger volume predictive at the first and second attempts [13]; larger volume in successful first attempts (8.7 ± 2.8 vs. 6.8 ± 3.0 mL) [40]; smaller volume in successful redo patients (8.2 ± 5.4 vs. 11.3 ± 5.3 mL, *p* = 0.01) [41]	The inverse association is confounded by KS, which accounted for 7 out of 23 successful retrievals [41]; volume alone neither predicts nor precludes success
Inter-procedure interval	Longer interval favourable, at the second attempt only	1.3-fold increase in odds per additional month [13]; 6–12 months commonly advised [1,4,11]	Intervals under six months feasible in selected men [28]; female partner age and assisted reproduction scheduling may justify a shorter interval
Klinefelter syndrome	Inconsistent across series	7/14 (50%) at redo [41] vs. 3/18 (16.7%) in a KS-only cohort [23]; 9/15 (60%) after a positive and 6/20 (30%) after a negative first micro-TESE in a KS redo series [35]; more prevalent among failed repeats (27% vs. 5%, *p* = 0.031) [2]	Differences most likely reflect selection, prior hormonal optimization, and residual focal spermatogenesis; individualized counselling required
Y-chromosome microdeletion	First-attempt evidence; extrapolated to redo	First-attempt populations: complete AZFa or AZFb deletions essentially incompatible with retrieval; AZFc retrieval rates approximately 50–71% [1,5]. These findings are extrapolated to redo	Genetic screening before redo; evidence is not derived from dedicated redo cohorts, and AZFc rates are not redo-specific estimates

FSH, follicle-stimulating hormone; ICSI, intracytoplasmic sperm injection; KS, Klinefelter syndrome; AMH, anti-Müllerian hormone; SCOS, Sertoli cell-only syndrome; SRR, sperm retrieval rate.

**Table 3:** Characteristics and outcomes of redo sperm-retrieval reports after previous failure.

Author, Year	n/Population	Design	Interval	Hormonal Pretreatment	SRR	Reproductive Outcomes
Shiraishi et al., 2012 [53]	48; 28 treated, 20 untreated	Non-randomized comparative study	NV	hCG for 4–5 months; rhFSH added according to gonadotropin suppression	6/28 (21.4%) vs. 0/20	NR
Shinjo et al., 2013 [54]	20 redo patients	Mechanistic treatment cohort; retrospective record review	Mean 13 months	hCG for 4–5 months; rhFSH added in 9/20	3/20 (15.0%)	NR
Haliloglu et al., 2014 [23]	18; KS only	Retrospective series	NR	NR	3/18 (16.7%)	1 live birth
Shiraishi et al., 2016 [24]	21	Prospective, multicentre, single-arm	NV	hCG for 1 month, then hCG + rhFSH for 3 months	2/21 (9.5%)	1 live birth
Yücel et al., 2018 [22]	49	Retrospective, single-centre	Minimum 3 months	None between procedures	21/49 (42.8%, as reported)	NR
Jarvis et al., 2018 [25]	82 mapped; 24 positive; 15 underwent retrieval	Retrospective; FNA-guided retrieval *	To mapping: mean: 2.2 years; mapping to retrieval: mean: 7.5 months	10/15 received medical optimization or varicocele repair *	15/15 selected operated men; 15/82 (18.3%) of mapped cohort	9 couples followed: 6 pregnancies, 4 births, 1 miscarriage, 1 ectopic pregnancy
Salvarci and Balasar, 2019 [35]	20 prior failures within 35 s procedures; KS only	Retrospective; mixed prior success/failure	NR	No infertility medication	6/20 (30.0%) after failure; mixed cohort 15/35 (42.8%, as reported)	After prior failure: 2 pregnancies among 6 retrieval successes; 1 live birth
Spahovic et al., 2020 [4]	3 selected redo patients from 33 index cases	Retrospective; selected second-look subgroup	NR	FSH/menotropin stimulation for 3 months	1/3 (33.3%)	NR
Amer et al., 2020 [55]	40; 20 treated, 20 controls	Prospective case–control; index technique uncertain †	NV	Testosterone downregulation for 1 month, then gonadotropins for 3 months	2/20 (10.0%) vs. 0/20	NR
Özman et al., 2021 [41]	125; 42 hormonally optimized	Retrospective series	NV	Anastrozole or clomiphene, with hCG if required	23/125 (18.4%); treated 11/42 vs. untreated 12/83	NR

Abbreviations: FNA, fine-needle aspiration; FSH, follicle-stimulating hormone; hCG, human chorionic gonadotropin; KS, Klinefelter syndrome; rhFSH, recombinant human FSH; SRR, sperm retrieval rate. NR, not reported in the available study description; NV, interval not verified from accessible primary-source information. Interval denotes time between retrieval procedures unless otherwise specified; treatment duration is not a substitute for this interval. * Jarvis et al. used FNA-guided TESA, TESE or micro-TESE; the 100% retrieval rate applies only to the 15 selected men who proceeded to retrieval. Medical optimization included anastrozole, clomiphene, FSH or hCG; the reported 10/15 also includes varicocele repair. Year follows the online publication cited in the bibliography. † The index c-TESE versus micro-TESE technique in Amer et al. was not clearly established in the evidence available for this review. Salvarci and Balasar’s prior-failure subgroup is distinguished from their mixed second-procedure cohort. Potential overlap among related reports has not been excluded; sample sizes and outcomes should not be summed or pooled. Missing outcomes do not imply zero pregnancies or births.

## Data Availability

No new data were created or analysed in this study. Data sharing is not applicable to this article.

## Supplementary Materials

The following supporting information can be downloaded at https://www.mdpi.com/article/10.3390/jcm15208088/s1.

## References

[1] Flannigan R., Bach P.V., Schlegel P.N. (2017). Microdissection testicular sperm extraction. Transl. Androl. Urol..

[2] Alriyalat S., Deameh M.G., Farraj H., Alsmadi J.K., Bani Irshid B.A., Abu Rahmeh H., Khaled Awawdeh A., Ahmad Mubarak M., Fawzey Ababneh A., Bani Amer W.A. (2025). Predictors of sperm retrieval success in first-time and repeated micro-TESE for nonobstructive azoospermia. Future Sci. OA.

[3] Elbardisi H., Bakircioglu E., Liu W., Katz D. (2025). Second chance in fertility: A comprehensive narrative review of redo micro-TESE outcomes after initial failure. Asian J. Androl..

[4] Spahovic H., Alic J., Göktolga Ü., Lepara Z., Lepara O., Rama A., Suljevic I. (2020). “Second-look” micro testicular sperm extraction (microTESE) in patients with non-obstructive azoospermia following histopathological analysis. Med. Arch..

[5] Stahl P.J., Masson P., Mielnik A., Marean M.B., Schlegel P.N., Paduch D.A. (2010). A decade of experience emphasizes that testing for Y microdeletions is essential in American men with azoospermia and severe oligozoospermia. Fertil. Steril..

[6] Jensen S., Ko E.Y. (2021). Varicocele treatment in non-obstructive azoospermia: A systematic review. Arab J. Urol..

[7] Schlegel P.N. (1999). Testicular sperm extraction: Microdissection improves sperm yield with minimal tissue excision. Hum. Reprod..

[8] Esteves S.C. (2022). Microdissection TESE versus conventional TESE for men with nonobstructive azoospermia undergoing sperm retrieval. Int. Braz. J. Urol..

[9] Corona G., Minhas S., Giwercman A., Bettocchi C., Dinkelman-Smit M., Dohle G., Fusco F., Kadioglou A., Kliesch S., Kopa Z. (2019). Sperm recovery and ICSI outcomes in men with non-obstructive azoospermia: A systematic review and meta-analysis. Hum. Reprod. Update.

[10] Bernie A.M., Mata D.A., Ramasamy R., Schlegel P.N. (2015). Comparison of microdissection testicular sperm extraction, conventional testicular sperm extraction, and testicular sperm aspiration for nonobstructive azoospermia: A systematic review and meta-analysis. Fertil. Steril..

[11] Wang X., Ying Z., Guo H., Zhang X., Jiang D. (2026). Micro-TESE in men with non-obstructive azoospermia: A narrative review of sperm retrieval benefit, testicular injury, and endocrine risk. Middle East Fertil. Soc. J..

[12] Zachariou A., Giannakis I., Baltogiannis D., Kaltsas A., Zikopoulos A., Stavros S., Kojovic V., Pantou A., Takenaka A., Sofikitis N. (2025). Understanding male sexual function after TESE or mTESE: A narrative review. Integrative Male Reproductive Health—Risk, Mechanisms, and Interventions.

[13] Ghalayini I.F., Alazab R., Halalsheh O., Al-Mohtaseb A.H., Al-Ghazo M.A. (2022). Repeated microdissection testicular sperm extraction in patients with non-obstructive azoospermia: Outcome and predictive factors. Arab J. Urol..

[14] Talas H., Yaman O., Aydos K. (2007). Outcome of repeated micro-surgical testicular sperm extraction in patients with non-obstructive azoospermia. Asian J. Androl..

[15] Friedler S., Raziel A., Schachter M., Strassburger D., Bern O., Ron-El R. (2002). Outcome of first and repeated testicular sperm extraction and ICSI in patients with non-obstructive azoospermia. Hum. Reprod..

[16] Kamal A., Fahmy I., Mansour R., Abou-Setta A., Serour G., Aboulghar M. (2004). Outcome of repeated testicular sperm extraction and ICSI in patients with non-obstructive azoospermia. Middle East Fertil. Soc. J..

[17] Westlander G., Rosenlund B., Söderlund B., Wood M., Lundin K., Hillensjö T. (2001). Sperm retrieval, fertilization, and pregnancy outcome in repeated testicular sperm aspiration. J. Assist. Reprod. Genet..

[18] Vernaeve V., Verheyen G., Goossens A., Van Landuyt L., Van Steirteghem A., Tournaye H. (2006). How successful is repeat testicular sperm extraction in patients with azoospermia?. Hum. Reprod..

[19] Tournaye H., Verheyen G., Nagy P., Ubaldi F., Goossens A., Silber S., Van Steirteghem A.C., Devroey P. (1997). Are there any predictive factors for successful testicular sperm recovery in azoospermic patients?. Hum. Reprod..

[20] Al-Zubi M., Al-Khawaldeh S., Mallak M., Al-Dghaim M., Zytoon R., Abbas R., Alhabahbeh A., Alfadel M., Abuorouq S., Al-Magableh M.R. (2025). Can we predict the outcome of micro testicular sperm extraction in non-obstructive azoospermia from preoperative hormonal profile, testicular volume, and patients’ health factors: A retrospective cross-sectional study. Am. J. Men’s Health.

[21] Kavoussi P.K., West B.T., Chen S.-H., Hunn C., Gilkey M.S., Machen G.L., Kavoussi K.M., Esqueda A., Wininger J.D., Kavoussi S.K. (2020). A comprehensive assessment of predictors of fertility outcomes in men with non-obstructive azoospermia undergoing microdissection testicular sperm extraction. Reprod. Biol. Endocrinol..

[22] Yücel C., Budak S., Keskin M.Z., Kisa E., Kozacioglu Z. (2018). Predictive factors of successful salvage microdissection testicular sperm extraction (mTESE) after failed mTESE in patients with non-obstructive azoospermia: Long-term experience at a single institute. Arch. Ital. Urol. Androl..

[23] Haliloglu A.H., Tangal S., Gulpinar O., Onal K., Pabuccu R. (2014). Should repeated TESE be performed following a failed TESE in men with Klinefelter syndrome?. Andrology.

[24] Shiraishi K., Ishikawa T., Watanabe N., Iwamoto T., Matsuyama H. (2016). Salvage hormonal therapy after failed microdissection testicular sperm extraction: A multi-institutional prospective study. Int. J. Urol..

[25] Jarvis S., Yee H.K., Thomas N., Cha I., Prasad K.C., Ramsay J.A. (2018). Sperm fine-needle aspiration (FNA) mapping after failed microdissection testicular sperm extraction (TESE): Location and patterns of found sperm. Asian J. Androl..

[26] Zhang F., Dai M., Yang X., Cheng Y., Ye L., Huang W., Chen X., Yin T., Sha Y. (2024). Predictors of successful salvage microdissection testicular sperm extraction (mTESE) after failed initial TESE in patients with non-obstructive azoospermia: A systematic review and meta-analysis. Andrology.

[27] Boeri L., Bebi C., Dente D., Greco E., Turetti M., Capece M., Cocci A., Cito G., Preto M., Pescatori E. (2022). Outcomes and predictive factors of successful salvage microdissection testicular sperm extraction (mTESE) after failed classic TESE: Results from a multicenter cross-sectional study. Int. J. Impot. Res..

[28] Tai M.C., Huang I.S., Huang C.Y., Huang W.J. (2023). Feasibility of repeat microdissection testicular sperm extraction within 6 months for nonobstructive azoospermia. J. Chin. Med. Assoc..

[29] Amer M.K., Ahmed A.R., Abdel Hamid A.A., GamalEl Din S.F. (2019). Factors determining the sperm retrieval rate in fresh versus salvage micro-TESE: A comparative cohort study. Int. Urol. Nephrol..

[30] Alahmari A., Akram R., Maalouf M., Elsayed A., Zini A. (2025). Redo testicular sperm aspiration (TESA) in men with severe oligoasthenoteratozoospermia (OAT) and obstructive azoospermia (OA). Can. J. Urol..

[31] Ryzhkov A., Rashed A., Arafa M., Pinggera G.-M., Wyns C., Le T.V., Falcone M., Shatylko T., Colpi G.M., Ramsay J.A. (2026). Management of non-obstructive azoospermia: Practical answers to key clinical questions. World J. Men’s Health.

[32] Haimov-Kochman R., Losso F., Nefesh I., Zentner B.-S., Moz Y., Prus D., Bdolah Y., Hurwitz A. (2009). The value of repeat testicular sperm retrieval in azoospermic men. Fertil. Steril..

[33] Corona G., Pizzocaro A., Lanfranco F., Garolla A., Pelliccione F., Vignozzi L., Ferlin A., Foresta C., Jannini E.A., Maggi M. (2017). Sperm recovery and ICSI outcomes in Klinefelter syndrome: A systematic review and meta-analysis. Hum. Reprod. Update.

[34] Kaltsas A., Stavros S., Kratiras Z., Zikopoulos A., Machairiotis N., Potiris A., Dimitriadis F., Sofikitis N., Chrisofos M., Zachariou A. (2024). Predictors of successful testicular sperm extraction: A new era for men with non-obstructive azoospermia. Biomedicines.

[35] Salvarci A., Balasar M. (2019). What should be the minimum frequency of micro testicular sperm extraction (m-TESE) in patients with Klinefelter syndrome?. Ann. Med. Res..

[36] Kaltsas A., Markou E., Zachariou A., Dimitriadis F., Symeonidis E.N., Zikopoulos A., Mamoulakis C., Tien D.M.B., Takenaka A., Sofikitis N. (2023). Evaluating the predictive value of diagnostic testicular biopsy for sperm retrieval outcomes in men with non-obstructive azoospermia. J. Pers. Med..

[37] Ramasamy R., Lin K., Gosden L.V., Rosenwaks Z., Palermo G.D., Schlegel P.N. (2009). High serum FSH levels in men with nonobstructive azoospermia do not affect success of microdissection testicular sperm extraction. Fertil. Steril..

[38] Pozzi E., Corsini C., Belladelli F., Bertini A., Negri F., Raffo M., Saccà A., Ventimiglia E., Boeri L., Fallara G. (2024). Role of follicle-stimulating hormone, inhibin B, and anti-Müllerian hormone in predicting sperm retrieval from men with nonobstructive azoospermia undergoing microdissection testicular sperm extraction: A systematic review and meta-analysis. Eur. Urol. Open Sci..

[39] Amer M.K., Zohdy W., GamalEl Din S.F., Moawad H.H., Hasan El Saedy D.A., Zaki Gamal E.A., Ragab A. (2023). Evaluation of the utility of seminal plasma resistin and leptin in predicting successful surgical sperm retrieval in men with non-obstructive azoospermia. J. Reprod. Infertil..

[40] Mohamed O., Hafez H., Tammam O., Alsagheer G.A., Hasan A. (2026). Predictive factors of successful microdissection testicular sperm extraction for patients with non-obstructive azoospermia. Afr. J. Urol..

[41] Özman O., Tosun S., Bayazıt N., Cengiz S., Bakırcıoğlu M.E. (2021). Efficacy of the second micro-testicular sperm extraction after failed first micro-testicular sperm extraction in men with nonobstructive azoospermia. Fertil. Steril..

[42] McLachlan R.I., Rajpert-De Meyts E., Hoei-Hansen C.E., de Kretser D.M., Skakkebaek N.E. (2007). Histological evaluation of the human testis: Approaches to optimizing the clinical value of the assessment: Mini review. Hum. Reprod..

[43] Johnsen S.G. (1970). Testicular biopsy score count: A method for registration of spermatogenesis in human testes, normal values and results in 335 hypogonadal males. Hormones.

[44] Levin H.S. (1979). Testicular biopsy in the study of male infertility: Its current usefulness, histologic techniques, and prospects for the future. Hum. Pathol..

[45] Ramasamy R., Padilla W.O., Osterberg E.C., Srivastava A., Reifsnyder J.E., Niederberger C., Schlegel P.N. (2013). A comparison of models for predicting sperm retrieval before microdissection testicular sperm extraction in men with nonobstructive azoospermia. J. Urol..

[46] Ramasamy R., Schlegel P.N. (2007). Microdissection testicular sperm extraction: Effect of prior biopsy on success of sperm retrieval. J. Urol..

[47] Gao S., Yang X., Xiao X., Yin S., Guan Y., Chen J., Chen Y. (2022). Outcomes and affecting factors for ICSI and microTESE treatments in nonobstructive azoospermia patients with different etiologies: A retrospective analysis. Front. Endocrinol..

[48] Krausz C., Navarro-Costa P., Wilke M., Tüttelmann F. (2024). EAA/EMQN best practice guidelines for molecular diagnosis of Y-chromosomal microdeletions: State of the art 2023. Andrology.

[49] Aoki S., Takeshima T., Mimura N., Seki H., Yumura Y. (2025). Successful sperm retrieval by microdissection testicular sperm extraction in a man with partial AZFb deletion: A case report. Transl. Androl. Urol..

[50] Eliveld J., van Wely M., Meißner A., Repping S., van der Veen F., van Pelt A.M.M. (2018). The risk of TESE-induced hypogonadism: A systematic review and meta-analysis. Hum. Reprod. Update.

[51] Tharakan T., Corona G., Foran D., Salonia A., Sofikitis N., Giwercman A., Krausz C., Yap T., Jayasena C.N., Minhas S. (2022). Does hormonal therapy improve sperm retrieval rates in men with non-obstructive azoospermia: A systematic review and meta-analysis. Hum. Reprod. Update.

[52] Shiraishi K. (2015). Hormonal therapy for non-obstructive azoospermia: Basic and clinical perspectives. Reprod. Med. Biol..

[53] Shiraishi K., Ohmi C., Shimabukuro T., Matsuyama H. (2012). Human chorionic gonadotrophin treatment prior to microdissection testicular sperm extraction in non-obstructive azoospermia. Hum. Reprod..

[54] Shinjo E., Shiraishi K., Matsuyama H. (2013). The effect of human chorionic gonadotropin-based hormonal therapy on intratesticular testosterone levels and spermatogonial DNA synthesis in men with non-obstructive azoospermia. Andrology.

[55] Amer M.K., Ahmed H.E., GamalEl Din S.F., Fawzy Megawer A., Ahmed A.R. (2020). Evaluation of neoadjuvant gonadotropin administration with downregulation by testosterone prior to second time microsurgical testicular sperm extraction: A prospective case-control study. Urologia.

[56] Laursen R.J., Alsbjerg B., Elbaek H.O., Povlsen B.B., Jensen K.B.S., Lykkegaard J., Esteves S.C., Humaidan P. (2022). Recombinant gonadotropin therapy to improve spermatogenesis in nonobstructive azoospermic patients: A proof of concept study. Int. Braz. J. Urol..

[57] Aydos K., Ünlü C., Demirel L.C., Evirgen O., Tolunay O. (2003). The effect of pure FSH administration in non-obstructive azoospermic men on testicular sperm retrieval. Eur. J. Obstet. Gynecol. Reprod. Biol..

[58] European Association of Urology EAU Guidelines on Sexual and Reproductive Health: Male Infertility. https://uroweb.org/guidelines/sexual-and-reproductive-health/chapter/male-infertility.

[59] Takalani N.B., Monageng E.M., Mohlala K., Monsees T.K., Henkel R., Opuwari C.S. (2023). Role of oxidative stress in male infertility. Reprod. Fertil..

[60] Dutta S., Sengupta P., Slama P., Roychoudhury S. (2021). Oxidative stress, testicular inflammatory pathways, and male reproduction. Int. J. Mol. Sci..

[61] Smits R.M., Mackenzie-Proctor R., Yazdani A., Stankiewicz M.T., Jordan V., Showell M.G. (2019). Antioxidants for male subfertility. Cochrane Database Syst. Rev..

[62] Esteves S.C., Miyaoka R., Roque M., Agarwal A. (2016). Outcome of varicocele repair in men with nonobstructive azoospermia: Systematic review and meta-analysis. Asian J. Androl..

[63] Kaltsas A., Markou E., Zachariou A., Dimitriadis F., Mamoulakis C., Andreadakis S., Giannakis I., Tsounapi P., Takenaka A., Sofikitis N. (2022). Varicoceles in men with non-obstructive azoospermia: The dilemma to operate or not. Front. Reprod. Health.

[64] Barda S., Fuchs Weizman N., Hauser R., Aspitz H.Z., Amir H., Kalma Y., Lehavi O., Kleiman S.E., Azem F., Dekalo S. (2026). Combining microTESE and trifocal TESE improves sperm retrieval and cryopreservation outcomes in nonobstructive azoospermia. Int. Urol. Nephrol..

[65] Marconi M., Keudel A., Diemer T., Bergmann M., Steger K., Schuppe H.-C., Weidner W. (2012). Combined trifocal and microsurgical testicular sperm extraction is the best technique for testicular sperm retrieval in “low-chance” nonobstructive azoospermia. Eur. Urol..

[66] Almajed W., Alharbi M., Zini A. (2020). Use of mini-incision microdissection testicular sperm extraction in men with cryptozoospermia and non-obstructive azoospermia. Andrology.

[67] Beliveau M.E., Turek P.J. (2011). The value of testicular ‘mapping’ in men with non-obstructive azoospermia. Asian J. Androl..

[68] Gnessi L., Scarselli F., Minasi M.G., Mariani S., Lubrano C., Basciani S., Greco P.F., Watanabe M., Franco G., Farcomeni A. (2018). Testicular histopathology, semen analysis and FSH, predictive value of sperm retrieval: Supportive counseling in case of reoperation after testicular sperm extraction (TESE). BMC Urol..

[69] Achermann A.P.P., Pereira T.A., Esteves S.C. (2021). Microdissection testicular sperm extraction (micro-TESE) in men with infertility due to nonobstructive azoospermia: Summary of current literature. Int. Urol. Nephrol..

[70] Yu Z., Wei Z., Yang J., Wang T., Jiang H., Li H., Tang Z., Wang S., Liu J. (2018). Comparison of intracytoplasmic sperm injection outcome with fresh versus frozen-thawed testicular sperm in men with nonobstructive azoospermia: A systematic review and meta-analysis. J. Assist. Reprod. Genet..

