# Supplementary material for: Repeat and Redo Microdissection Testicular Sperm Extraction (Micro-TESE) in Non-Obstructive Azoospermia: A Narrative Review
Source: J Clin Med. 2026 Oct 18;15(20):8088. doi: 10.3390/jcm15208088 (PMC13706044; doi:10.3390/jcm15208088)
Supplement: Supplementary file 1 [file jcm-15-08088-s001.zip › jcm-4563004-supplementary.pdf]

Supplementary Table S1

SANRA assessment of the narrative review

Manuscript: Repeat and Redo Microdissection Testicular Sperm Extraction (Micro-TESE) in Non-Obstructive Azoospermia: A Narrative Review. Version assessed: 1n.

Status: provisional editorial assessment prepared during manuscript revision, requiring author review. This is not an independent assessment by the author team. Scores evaluate what is reported in the manuscript; they do not verify unreported search or screening procedures.

Each item is scored from 0 to 2; the maximum total is 12. The assessment below applies the SANRA domains with manuscript-specific explanations.

SANRA domain	0–2	Location	Basis for assessment
1. Importance to readers	2	Introduction and Sections 6–7	Explains the clinical need for further sperm retrieval, the distinction between repeat and redo procedures, and the risks and counselling implications.
2. Explicit review aims	2	Final paragraph of Introduction	Specifies retrieval outcomes, prognostic factors, and hormonal and antioxidant optimization before further surgery.
3. Search description	1	Section 2	Databases, date window, concepts, eligibility criteria, and citation chasing are described. Exact reproducible search strings, search-flow totals, and author responsibilities remain incomplete. Score is deliberately provisional.
4. Referencing	2	Throughout; References; Tables 1–2	Numerical claims are linked to identifiable sources and the bibliography follows first-citation order. This score does not constitute an independent full-text verification of every citation.
5. Scientific reasoning	2	Sections 3–7; figure legends	Distinguishes prior success from prior failure, identifies selection and cohort heterogeneity, separates direct evidence from extrapolation, and discusses uncertainty and non-randomized designs.
6. Relevant outcome data	2	Sections 3–6; Tables 1–2	Reports denominators, retrieval and reproductive outcomes, comparative estimates, endocrine decline, and study limitations; cautions against treating selected repeat cohorts as an unselected prognosis.

Provisional total: 11/12. The total is not a certification of methodological validity or completeness. Reassess after the outstanding Methods information is supplied.

Source: Baethge, C.; Goldbeck-Wood, S.; Mertens, S. SANRA—a scale for the quality assessment of narrative review articles. Research Integrity and Peer Review 2019, 4, 5. https://doi.org/10.1186/s41073-019-0064-8.
